# The Influence of Continuous Improvement of Public Car-Sharing Platforms on Passenger Loyalty: A Mediation and Moderation Analysis

**DOI:** 10.3390/ijerph17082756

**Published:** 2020-04-16

**Authors:** Fei Ma, Dan Guo, Kum Fai Yuen, Qipeng Sun, Fuxia Ren, Xiaobo Xu, Chengyong Zhao

**Affiliations:** 1 School of Economics and Management, Chang’an University, Xi’an 710064, China; mafeixa@chd.edu.cn (F.M.); sunqip@chd.edu.cn (Q.S.); 2018123034@chd.edu.cn (F.R.); 2016903877@chd.edu.cn (C.Z.); 2 Youth Innovation Team of Shaanxi Universities, Chang’an University, Xi’an 710064, China; 3 School of Civil and Environmental Engineering, Nanyang Technological University, Singapore 639798, Singapore; kumfai.yuen@ntu.edu.sg; 4 School of Business Administration, American University of Sharjah, Sharjah 26666, UAE; xiaobo@aus.edu

**Keywords:** car-sharing, continuous improvement, passenger loyalty, structural equation model, perceived value

## Abstract

Public car-sharing is a growing business model that contributes to sustainable transportation and urban development. The continuous improvement of public car-sharing platform to garner passenger loyalty is vital for a car-sharing platform’s success. This study applied perceived value theory, trust theory, and transaction cost theory to construct a structural equation model in order to explain passenger loyalty. Data from 755 surveys were collected using stratified sampling in mainland China. The estimated results of the theoretical model show that the relationship between continuous improvement and passenger loyalty is mediated by passenger perceived value, passenger trust, and transaction costs. Consequently, a multi-group analysis is conducted to analyze the moderation effects of passenger’s license and car-sharing experience on the theoretical model. The results show that some of the path coefficients are significantly different between these sub-groups. This indicates that platforms should provide differentiate services for passengers based on the purpose of using car-sharing and usage experience. This study provides new theoretical insights into understanding passenger loyalty with respect to public car-sharing and provides policy recommendations for the sustainable development of public car-sharing.

## 1. Introduction

Car-sharing has received increasing attention and use in recent years [1]. Shared cars and their operating systems are owned and maintained by car-sharing organizations [2]. Passengers can pick up, use, return, and pay for their cars using applications from public car-sharing platforms. Many researchers and practitioners have highlighted the great potential of car-sharing in the context of business opportunities and sustainable solutions [3,4,5,6,7]. Car sharing is expected to provide high vehicle utilization, minimal land use, significant cost savings, and substantial environmental potential and social benefits for future sustainable transport systems [8]. When the vehicles are installed with are Battery Electric Vehicles (BEV), car-sharing has been proven to ease the energy and environmental crisis [9]. However, Jung and Koo [10] found that giving up the purchase of cars could not offset the increase of greenhouse gas emissions that is caused by the transition from public transportation or private car use to car-sharing. The extent to which traffic congestion and carbon dioxide emissions are reduced depends on the type of shared traffic [11]. In fact, in any city, it is hard to determine how many trips can actually be replaced by shared mobile services [11].

Although the environmental benefits of car-sharing are not clear, car-sharing is indeed a missing link in sustainable transportation, combining the flexibility, mobility, and accessibility of private cars with the economics and sustainability of public transportation [12]. Mannan [13] believes that, to promote sustainable development, transport policy must address not only environmental issues, but also equity and economic sustainability. One solution is to build ring roads, subways, train tracks, trams, extra bus lines, and more bike lanes. Another approach is to use rich information technology in order to introduce a new generation of car-sharing systems. Car-sharing services are an important new business in the field of sustainable transportation [1], and have significantly grown, particularly in metropolitan areas [7]. Further, car sharing can bring profits to private companies [11].

Cohen and Kietzmann [14] pointed out that car sharing is aimed at individuals who intend to transfer ownership to a shared vehicle or share their own vehicle when not in use. Hui et al. [15] in a questionnaire survey of members of the “FunCarsharing” system in Hangzhou, China, showed that 36.4% of the respondents’ commuting mode is “Drive or ride a car”, and 32.1% of the respondents’ commuting traffic mode is “Transit”, and 2.6% of the respondents’ commuting mode is “Taxi”. “Bike or walk” and “Others” account for 25.4% and 3.7%, respectively. Therefore, car-sharing users can be private car owners, as well as users of taxis and public transport. Private car users may choose to use a shared car due to vehicle restrictions or different travel destinations between family members. Taxi users choose shared cars because they are cheaper than taxis. Public transport users choose it because it is more comfortable and convenient. When using a shared car, they can assume two identities: driver or rider. The group with the driver’s licenses refers to the people who drive shared cars; in contrast, the group without driver’s licenses refers to the people who use (ride) shared cars. The group without driver’s licenses uses a shared car when their family, friends, or possibly a ride-sharing online friend acts as the driver. Therefore, both drivers and riders are passengers (users).

Passengers and enterprises are the main participants of car-sharing, ensuring the implementation and development of the shared travel model [16]. Bi et al. [17] found that a car-sharing company in Beijing has 35,000 users, of which 39% are silent users and 61% are non-silent users. The silent users are defined as users who have orders in the first three months and no orders thereafter. It can be seen that there is a considerable loss of passengers on the public car-sharing platforms. This might be because there are many problems with the operation of the public car-sharing platforms, such as the lack of service capabilities (e.g. shared car placement area is limited or pick up and return of the car must be at the same operating point) and the need to explore new modes of public car-sharing. Therefore, the services of the public car-sharing platforms need constant maintenance, improvement and adjustment. It is important that the public car-sharing platforms continuously improves and adjusts its business to meet passenger expectations for car-sharing services. The passengers are the customers of car-sharing services. Therefore, public car-sharing platforms require users to use their service to support their scale of operations [16]. Their satisfaction and willingness to use car-sharing again are the focus of most public car-sharing platforms. As such, the public car-sharing platform companies should improve the experience of car-sharing passengers (users) and their loyalty through continuous improvement.

Continuous improvement of public car-sharing platforms is the key to its survival and development [18]. The public car-sharing platforms carry out continuous improvement activities to improve the processes and overall organizational performance [19]. The development of public car-sharing platforms is inseparable from continuous improvement, and continuous improvement of public car-sharing platforms supports and strengthens passenger loyalty. However, this study proposes that the continuous improvement of public car-sharing platforms does not directly affect passenger loyalty due to the intangibility and inseparability (the production and consumption of services are simultaneous) of the services. Continuous improvement affects passenger loyalty through mediation variables.

When studying passenger loyalty, scholars have mostly focused on service quality, passenger satisfaction, and usage scenarios. Mugion [20] found that the quality of service directly affects citizens’ willingness to use public transport more, and this will affect both the willingness of citizens to use less cars and the willingness to use more sustainable vehicles, such as car sharing. Peng [21] found that passenger satisfaction has a direct positive impact on passenger loyalty in urban rail transit services, and some rail transit service quality factors directly positively impact passenger loyalty. Chao [22] selected seven variables (environment and facilities, operational services and efficiency, emotional value, perceived value, expectation, satisfaction, and loyalty) and established a structural equation model in order to examine passenger loyalty to bus services. Mattia [23] found that attitudes, perceived behavioral controls, and subjective norms have a significant impact on the willingness to reuse free-floating car-sharing services in the future.

Continuous improvement is the core principle of total quality management [24]. The continuous improvement of the public car-sharing platforms comprehensively enhances the service value of shared cars. Service quality, usage scenarios, and passenger satisfaction are essentially passengers’ recognition of the value of shared car services. Hence, this study uses passenger perceived value as one of the antecedent variables for passenger loyalty. Studies have shown that the perceived value of public car-sharing is an important predictor of passenger loyalty [25]. Blackwell [26] proposed a value-loyalty model, where the passenger perceived value plays a decisive role in passengers’ reusing of shared cars. Sirohi [27] found that passenger perceived value is positively correlated with passenger loyalty intentions. Meanwhile, studies have found that passenger trust drives the evolution of passenger perceived value. Hongfei and Xiaofei [28] proposed that that passenger loyalty can only be nurtured by gaining passenger trust. There is a significant positive correlation between passenger trust and passenger loyalty [29]. In addition, passenger trust leads to a reduction in transaction costs, and the transaction costs are inversely related to the repetitive use of public car-sharing [30].

This study evaluates how the continuous improvement of public car-sharing platforms affects passenger loyalty, using three mediation variables: passenger perceived value, passenger trust, and transaction costs, based on the brief literature review above. The study focuses on the following two issues: (1) the influence of continuous improvement of the public car-sharing platforms on the passenger loyalty; and, (2) the role of passenger perceived value, passenger trust, and transaction costs of car-sharing in mediating the impact of continuous improvement on passenger loyalty. The study provides a theoretical basis to explain passenger loyalty in the context of public car-sharing and policy recommendations for the sustainable development of public car-sharing.

**Figure 1:**
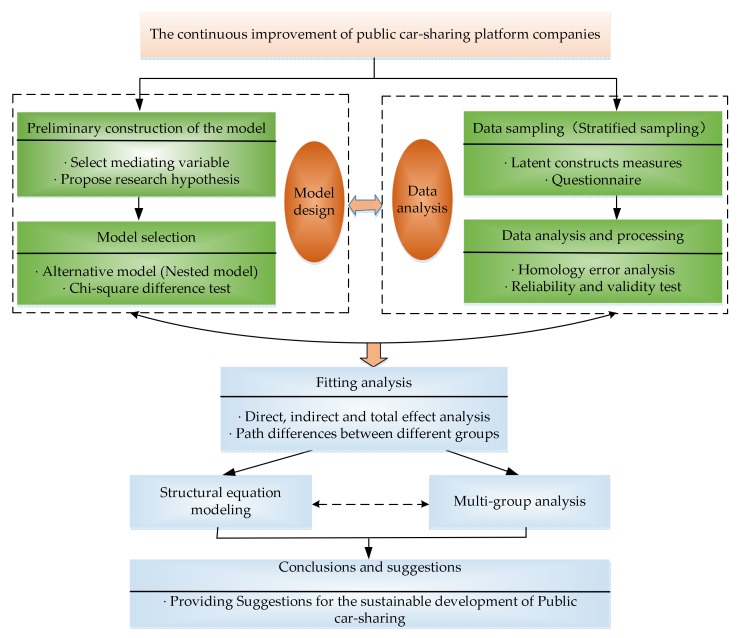
The research framework.

The rest of this paper is organized, as follows: Section 2 reviews the relevant literature on continuous improvement, perceived value theory, trust theory, and transaction cost theory; and, proposes the study’s theoretical model and research hypotheses. Section 3 describes the study’s research methods, including the measurement of latent variables, data collection, and collation. Section 4 applies structural equation modeling to verify the theoretical model and discuss the research results. Section 5 summarizes the research and proposes policy recommendations. Figure 1 illustrates the framework of this study.

## 2. Literature Review and Theoretical Model

### 2.1. Car-Sharing

Previous studies have conducted extensive analysis of car-sharing systems, which are mainly divided into two parts [31]. One part assesses their impact on transportation systems. Many researchers have analyzed the social, economic, and environmental impacts of car sharing [32,33,34]. These impacts include reduced emissions, fewer private cars, shorter driving distances, and increased mobility [10]. Another part is to study factors that influence people to choose car sharing services. Müller [35] uses perceived usefulness, perceived ease of use, attitude towards using, and behavioral intention to study the technical acceptance of car sharing. Empirical results show that perceived usefulness and perceived ease of use have a positive impact on attitude towards using, and attitudes have a positive effect on behavioral intentions. Tran et al. [36] uses an extended version of the Unified Theory of Technology and Acceptance (UTAUT) to study Chinese travelers’ acceptance of Electric Carsharing Systems (ECS). Performance expectancy, effort expectancy, social influence, familiarity with ECS, and hedonistic motivation are included in their model.

Different from previous research on car-sharing, we study the impact of continuous improvement of public car-sharing platforms on passenger loyalty. The use of mediation variables is required because this effect is indirect. Based on the literature review, we selected passenger perceived value, passenger trust, and transaction costs as mediation variables.

### 2.2. Continuous Improvement

Continuous improvement is a management philosophy that was adopted by companies to constantly improve one or some operational processes to increase customer satisfaction. Bessant and Caffyn [24] noted that continuous improvement involves a company-wide process that is more conducive and sustainable than radical innovations such as technology. Many other papers have cited the ideas of Bassant and his colleagues regarding continuous improvement. Those studies have focused on several issues. For example, the first group of research has focused on the strategic management of continuous improvement. They have investigated key areas for continuous improvement, and the effective ways to engage in the continuous improvement process. Klrner [37] reviewed the continuous improvement strategies used by various industries and examined their effectiveness under different industries.

The second group of studies has focused on factors that influence the continuous improvement of an organization. For instance, Sabater [38] interviewed first-line suppliers who have made continuous improvements in the automotive industry, identifying the drivers and inhibitors of continuous improvement. Yang [39] found that the employee’s awareness of continuous improvement has a regulatory effect on the decision-making process of continuous improvement activities in the continuous improvement preparation phase and it has a regulatory effect on the continuous improvement activity process in the continuous improvement implementation phase. In another study, Hu [40] found that the standardization of continuous improvement processes has a greater impact on continuous improvement measures.

The third research area focuses on the impact of continuous improvement on organizational performance. Continuous improvement has a positive impact on business operations, and companies around the world are implementing continuous improvement activities [41]. For example, Kumar [42] used continuous improvement and quality control technology to provide Mindarika Company with solutions and procedures for continuously improving its data management and product quality. Dabhilkar [43] confirmed the effectiveness of continuous improvement of behavioral evolution models in the results of the second international continuous improvement survey in Sweden and found that the development of continuous improvement capabilities helps to improve plant performance. Singh [44] found that continuous improvement increased the overall equipment efficiency of auto parts manufacturing plants by an average of 4.15%.

There have been many other studies showing that the continuous improvement of enterprises helps to improve enterprise competitiveness. Kovach and Fredendall [45] argued that continuous improvement efforts increase an employee’s understanding of the work environment; personal learning translates into organizational improvement.

Passenger loyalty reflects organizational performance for public car-sharing platforms. The public car-sharing platforms are in the development stage, and the functions of the platforms remain incomplete, with significant room for improvement. Through continuous improvement, public car-sharing platforms can gradually optimize service process, improve service quality and efficiency, and enrich the passenger service experience. The process of continuous improvement is expected to strengthen the service capacity of public car-sharing platforms. This should help to increase the retention rate of car-sharing users while attracting more new users. Therefore, this study proposes that the continuous improvement of public car-sharing platforms affects the loyalty of the shared car users.

### 2.3. Perceived Value Theory

Customer perceived value is a customer’s subjective evaluation of the utility of a product or service after deducting the cost of the product or service. In early studies, customer perceived value was conceptualized as a one-dimensional structure, which was derived from the evaluation of benefits and sacrifices that are related to products or services [46]. Customer perceived value varies with the place of purchase (consumption) and time of purchase (consumption). In general, customer perceived value includes four aspects: economic (e.g., price), functional (e.g., quality or performance), emotional (e.g., feelings and emotions), and social utility (e.g., self-concept) [47]. Hence, this study uses the definition of the multi-dimensional structure. Customers make purchase decisions based on perceived value. Passengers may continue to buy products or services of high perceived value and might even influence their subsequent behavioral intentions, including recommendations to others [48]. Parasuraman [49] found that customer perceived value had a direct and decisive influence on the customer’s willingness to repeatedly purchase a good. Some studies have confirmed that customer perceived value is related to customer loyalty. An empirical study by Mcdougall et al. [50] found that customer perceived value positively affected customer loyalty. Wu et al. [51] reported that customer perceived value and repurchase intention were positively correlated in the e-commerce environment from the perspective of online shoppers.

In the context of public car-sharing, the continuous improvement of the platforms could continuously create value. Public car-sharing platforms create value for passengers and improve their experience with and recognition of car-sharing by emphasizing brand marketing and service quality.

#### 2.3.1. Continuous Improvement and Passenger Perceived Value

Public car-sharing platforms can constantly improve the application’s interface design, optimize software, and usage processes, and reduce the waiting time of passengers. These improvements can increase passenger perceived value of the car-sharing service. At the same time, the continuous improvement of public car-sharing platforms requires employees to constantly learn and improve their daily tasks and performance [52]. Therefore, passengers will perceive higher service value when interacting with the employees. Hence, we state the following hypothesis:

 **H1:**  *The continuous improvement of public car-sharing platforms has a positive impact on passenger perceived value.*

#### 2.3.2. Passenger Perceived Value and Passenger Loyalty

Passenger perceived value is the basis of passenger loyalty. The service value that is provided by public car-sharing platforms to passengers is a key factor in increasing passenger loyalty [53]. Passenger perceived value has a positive impact on a passenger’s choice to reuse a car-sharing service and recommend others to use shared cars [54]. Studies have shown that passenger perceived value directly affects a passenger’s attitude loyalty (repeat purchase intention) and passenger behavioral loyalty (repeat purchase) [55]. Therefore, we make the following hypothesis:

 **H2:**  *The passenger perceived value of public car-sharing has a positive impact on passenger loyalty.*

### 2.4. Trust Theory

Trust is an abstract concept in sociological research, which has been defined by scholars from different perspectives without consensus [56]. Moorman et al. [57] believe that consumer trust, which refers to consumers’ confidence in the integrity, honesty, willingness to cooperate, reliability and comfort of the company, and personal trust in the company, is key to building customer relationships [58]. Meanwhile, some research shows that customer satisfaction is the antecedent of trust [59]. A continuous positive experience delivered to passengers who use public car-sharing increases their dependence on it, gradually forming satisfaction, which turns into trust. In essence, passenger trust is formed by the accumulation of passenger perceived value.

#### 2.4.1. Passenger Perceived Value and Passenger Trust

The consistent portrayal of service value in repeated transactions forms the basis for passenger trust [60]. Research shows that customer perceived value has a positive impact on customer trust. Trust has a strong influence on customer loyalty and it plays an important role in influencing customer loyalty [61]. This leads to the following hypothesis:

 **H3:**  *Passenger perceived value has a positive impact on public car-sharing passenger trust.*

#### 2.4.2. Passenger Trust and Passenger Loyalty

Morgan and Hunt [62] argued that passenger trust is the passenger’s confidence in the reliability and integrity of public car-sharing platforms, which is a factor in generating passenger loyalty. In a public car-sharing service environment, passengers face greater purchase risks and uncertainties. Many passengers do not accept public car-sharing, because they are reluctant to provide personal information to the public car-sharing platforms and pay a higher deposit [63]. If public car-sharing platforms can build passenger trust, the risks that are perceived by passengers will be reduced and loyalty will be improved [64]. This leads to the following hypothesis:

 **H4:**  *Passenger trust in public car-sharing has a positive impact on passenger loyalty.*

### 2.5. Transaction Cost Theory

Transaction costs are the expenses paid by the people for voluntary exchanges and cooperation with each other. In essence, there are transaction costs that are associated with most human exchange activities, which makes them an inseparable part of human social life. Tate [65] described the transaction costs as including information acquisition costs, bargaining costs, and execution costs. Coase [66] proposed the concept of transaction fees, and he reported that the cost of negotiating contracts for each transaction occurring in the market must also be taken into account. Williamson [67] suggested that the transaction costs should include the cost of drafting, negotiating, and executing contracts, and the cost of the governance that ensures the contracts go smoothly. Rahman and Kumaraswamy [68] noted that the transaction costs increase if there is a breach in contract.

Scholars often study the transaction costs between enterprises; however, public car-sharing services also incur information acquisition costs, bargaining costs, and execution costs. Before using public car-sharing platforms, passengers collect different information, such as the price and type of products or services. There is no bargaining between customers and public car-sharing platforms; however, passengers will compare the service prices and service advantages of each platform. The transaction costs refer to a series of costs incurred when the passengers select the public car-sharing platform and accept the services that are provided by the public car-sharing platform. These may include the cost of picking up the car when the passenger goes to the shared car branch.

The diversity of travel modes leads passengers to compare and select different options. Passengers usually focus on cost performance. Therefore, valuable services are not sufficient; public car-sharing platforms should reduce the passenger costs as much as possible.

#### 2.5.1. Passenger Perceived Value and Transaction Costs

Passengers have to pay a cost to understand and experience public car-sharing services. Communicating service value to passengers can reduce the cost of discussing and boost passengers’ confidence in using the platform [69]. Public car-sharing platforms must promote their value to passengers through different marketing channels, to ensure that passengers are aware of and appreciate those values. This reduces the cost incurred by passengers to access information. When passengers perceive high value of public car-sharing, they will be more proactive in understanding and using shared cars. The psychological satisfaction and pleasure somewhat compensate for the transaction costs that are paid by passengers. This leads to the following hypothesis:

 **H5:**  *The perceived value of public car-sharing passengers has a negative impact on transaction costs.*

#### 2.5.2. Transaction Costs and Passenger Loyalty

Reducing transaction costs and increasing the availability of public car-sharing sites significantly impact passenger loyalty [70]. When the use of a shared car has high transaction costs, passengers may remain using private cars, taxis, or public transport. Rational passengers will continue to use shared cars if using shared cars results in the lowest opportunity cost. This leads to the following hypothesis:

 **H6:**  *The transaction costs of public car-sharing have a negative impact on passenger loyalty.*

#### 2.5.3. Passengers Trust and Transaction Costs

Passenger trust in public car-sharing platforms reduces the cost of searching for information. Most information is directly provided by the public car-sharing platform; as such, passengers do not need to spend time searching. In addition, passenger trust in public car-sharing platforms makes people believe that they received high-quality services, so they do not need to check and confirm service quality. Even if problems occur, passengers believe they will be quickly solved. This leads to the following hypothesis:

 **H7:**  *Public car-sharing passenger trust has a negative impact on transaction costs.*

**Figure 2:**
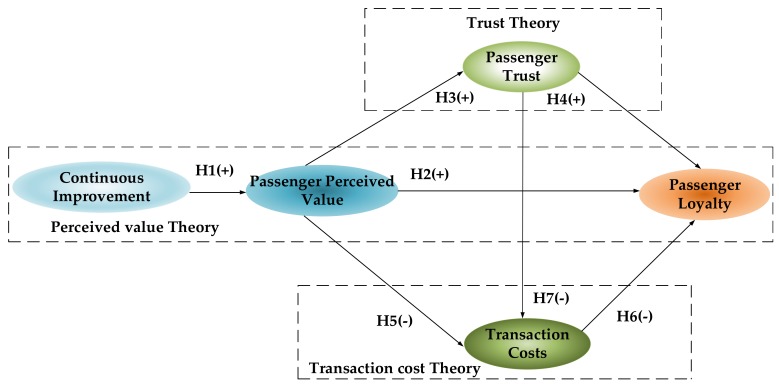
Theoretical model. Notes: (+) represents positive influence, (-) represents negative influence.

Based on the literature review above, combined with the theory of perceived value, trust theory, and transaction cost theory, we propose the theoretical model and hypotheses proposed in this study, as shown in Figure 2.

## 3. Research Methodology

### 3.1. Measures of Latent Constructs

Table 1 shows the latent constructs, measurement items, and the supporting literature for this study. The data were collected using questionnaires, designed using a five-level Likert scale, with 1 representing “strongly disagree” and 5 representing “strongly agree.”

The measurement of the continuous improvement (CI) of car-sharing platforms was based on studies conducted by Huang et al. [71] and Aloini et al. [72]. Public car-sharing platforms must continuously improve all aspects of products and processes [71]. Public car-sharing platforms continuously monitor, measure, and improve their activities. They also use passenger feedback to improve performance [72]. Based on this, this study developed six items to measure continuous improvement. Four measurement items were designed to measure passenger perceived value (PPV), based on economic value, functional value, emotional value, and social utility (see Table 1).

Five measurement programs were designed to measure passenger trust (PT) in the commitment of public car-sharing platform companies to continuously improve, and exhibit professionalism, integrity, and public welfare [69]. The measurement of transaction costs was borrowed from the practice of Tate [65] and it was modified based on the research background and environment.

Passengers may have differences, conflicts, disputes, order changes, and claims. These problems increase the transaction costs [73] during public car-sharing. Therefore, item TC4 was added in the measurement system, as the cost of ride-sharing generally exceeds the cost of a taxi (see Table 1). The measurement of passenger loyalty (PL) was drawn from the research of Zeithaml [74], as those measures have been shown to be valid under most contexts [69]. Loyal passengers should recognize the services of the car-sharing platform, communicate positive aspects of the car-sharing platform to friends and relatives, and encourage them to use the platform. Based on this, four measurement items were designed for measuring passenger loyalty (see Table 1).

**Table 1:** Construct, Measures, and Sources.

Construct	Measures	Adapted Source
Continuous Improvement	CI1. Public car-sharing platform companies make improvements in updating their vehicles.	Huang et al. [71] Aloini et al. [72]
	CI2. Public car-sharing platform companies continuously pay attention to the cleanliness of the interior of the vehicle and strive to keep the vehicle in good technical condition.	
	CI3. I did not experience vehicle battery power problems (or fuel shortage) during the use of the vehicle which has affected the travel situation.	
	CI4. When encounter problems using the car, public car-sharing platform companies address them in a more timely way.	
	CI5. Response rates and improvements in addressing customer complaints have improved.	
	CI6. After the driving trip, public car-sharing platforms conduct a timely follow-up with passengers and adopt their suggestions.	
Passengers Perceived Value	PPV1. The service pricing of public car-sharing platform companies is reasonable.	Zauner et al.[46] Sweeney and Soutar [75]
	PPV2. Continuous improvement of public car-sharing platform companies improves service performance (such as passenger driving comfort and safety, etc.).	
	PPV3. I was deeply impressed by the continuous improvement of the service of the public car-sharing platform companies.	
	PPV4. There is value in the continuous improvement of public car-sharing platform companies’ services.	
Passenger Trust	PT1. Public car-sharing platform companies can effectively and continuously improve their services.	Yuen et al. [69]
	PT2. Public car-sharing platform companies have the knowledge and skills needed to continuously improve their services.	
	PT3. Public car-sharing platform companies are truthful in their disclosure of continuous improvement information.	
	PT4. Public car-sharing platform companies sincerely continue to improve services.	
	PT5. The continuous improvement in the service provided by public car-sharing platform companies is oriented to meet the needs of the public, rather than self-interests.	
Transaction costs	TC1. I had to invest effort to collect information about the public platform companies before using the shared car.	Tate et al. [65]
	TC2. To use a shared car, I have to spend a lot of time in advance to understand the process.	
	TC3. I have to spend a lot of time learning about the process of handling public car-sharing accidents to prevent disputes after traffic accidents.	
	TC4. Generally speaking, the cost of using shared cars is higher compared to taxis.	
Passengers Loyalty	PL1. I think the shared car is my first choice for travel.	Zeithaml et al. [74]
	PL2. I will recommend the public car-sharing service of this platform company to my colleagues and friends.	
	PL3. I would encourage others to use the company’s car-sharing service.	
	PL4. I have positive comments on the service provided by the public car-sharing platform company.	

### 3.2. Stratified Sampling and Data Collection Methods

**Figure 3:**
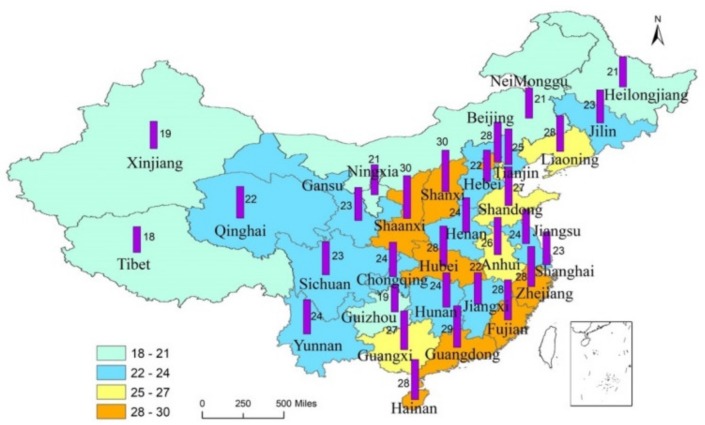
**Figure 3**. The stratified sampling region.

The study area included 31 provinces in mainland China. The questionnaire was distributed while using a stratified sampling method. The participants should have some understanding of the concept of car-sharing before answering the questionnaire in order to improve content validity [36]. An online questionnaire was sent to people who have used or know (have viewed information about shared cars) shared cars. A total of 850 questionnaires were collected from December 2018 to March 2019. There were 95 incomplete or short response questionnaires, leaving an effective count of 755 questionnaires (the questionnaire recovery rate was 88.82%). Figure 3 shows the distribution of questionnaires; each province was strongly represented. Table 2 shows that the sample is highly representative.

### 3.3. Common Method Bias Analysis, Reliability and Validity Test, Discriminant Validity Test

This research involves the collection of cross-sectional data. Therefore, common method bias, whereby the questionnaire is self-administered, might affect the research results. Data are subjected to Harman single factor test prior to data analysis [76]. The test results show that the variance of the first principal component generated while using the non-rotation factor analysis is 27.69% (less than 40%). This indicates that the common method bias was not significant.

The validity and reliability of the questionnaire was tested using confirmatory factor analysis and the Cronbach coefficient α, respectively. The KMO value is 0.900, which meets Kaiser’s [77] standard for factor analysis and passed the Bartlett test. Table 3 shows the results of the factor analysis, including the factor loading (*λ*), Cronbach coefficient (*α*), composite reliability (CR), and average variance extracted (AVE). The Cronbach coefficients in this study exceed the recommended 0.7 [78], which indicates that the study data have a high degree of reliability. CR values are above 0.8, exceeding the 0.7 that was recommended by Larcker [79]. The AVE values exceed 0.5, indicating that the data had high convergent validity.

The discrimination validity was evaluated by comparing the AVE value with the squared correlation coefficient [80]. In general, the squared correlations of the constructs are less than their AVEs. This condition is met, as shown in Table 4. Therefore, discriminant validity is supported.

## 4. Results and Discussion

### 4.1. Robustness Test of the Alternative Model

**Figure 4:**
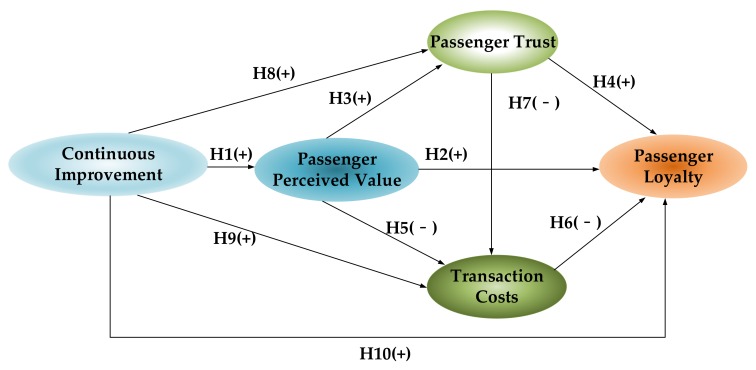
Alternative Model 1.

In constructing a structural equation model, the proposed theoretical model is not necessarily the most robust model. Therefore, two alternative models are proposed for further comparison. When compared to the theoretical model, in the alternative model 1 (in Figure 4), the continuous improvement directly affects the perceived value of passengers, passenger trust, transaction costs, and passenger loyalty.

The theoretical model and alternative models 1 and 2 are all nested models. As such, the chi-square difference test is used to compare the superiority of the model [81,82]. The chi-square difference test is used to compare the nested models in pairs, in the order of degrees of freedom, from low to high. When the comparison results are significant (*p* < 0.05), the more parsimonious model (the model with more degrees of freedom) is rejected, and the less parsimonious model (the model with fewer degrees of freedom) is accepted. When the comparison results are not significant (*p* > 0.05), the more parsimonious model is accepted, while the less parsimonious model is rejected.

Table 5 shows that alternative models 1 and 2 are compared first. The test results show that the chi-square distribution difference test (Δ *χ^2^* = 1.52, Δ *df* = 1) is not significant (*p* > 0.05). That is, the direct impact of continuous improvement of public car–sharing platforms on passenger loyalty does not affect model fit after being deleted. Therefore, the alternative model 1 is rejected and the alternative model 2 is accepted. The chi-square distribution difference test (Δ *χ^2^* = 2.92, Δ*df* = 2) of the alternative model 2 and the theoretical model is also not significant (*p* > 0.05). In other words, the model fit is not significantly affected when the direct impact of continuous improvement on passenger trust and transaction costs are deleted. Hence, alternative Model 2 is rejected, and the theoretical model is accepted.

**Figure 5:**
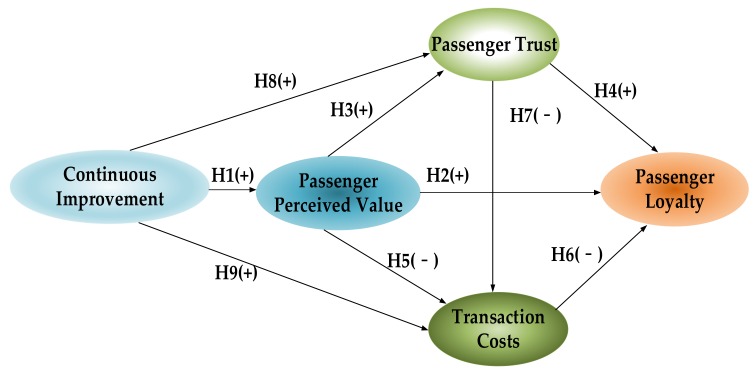
Alternative Model 2.

This leads to three additional paths in alternative model 1. The first path is H8, denoting that the continuous improvement positively impacts passenger trust. The second path is H9, denoting that the continuous improvement positively impacts transaction costs. The third path is H10, denoting that the ability of public car-sharing platforms to continuously improve has a direct positive impact on passenger loyalty. The above three paths all cross through passenger’s perceived value, which indicates a partial mediation relationship. Alternative model 2 (Figure 5) did not consider the direct relationship between continuous improvement and passenger loyalty, as compared to alternative model 1. Alternative model 2 proposes that the continuous improvement ability of public car-sharing platforms does not directly impact passenger loyalty, but it directly impacts passenger trust and transaction costs.

### 4.2. Theoretical Model Estimation

**Figure 6:**
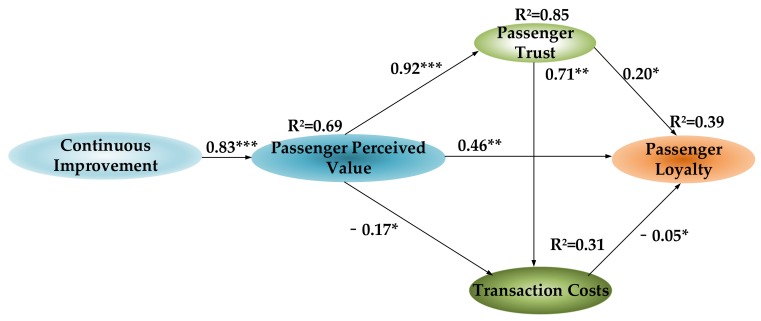
Parameter Estimation of the Theoretical Model.

The analysis shows that the theoretical model is the most robust of the three models. The parameters of the theoretical model are estimated while using the maximum likelihood function. Figure 6 shows the parameter estimates of the theoretical model and the coefficient of determination (*R^2^*) of the endogenous variables.

Model fit indices: *χ^2^* = 448（*p* = 0.000, *df* = 245; CFI = 0.962; TLI = 0.945; RMSEA = 0.060; SRMR = 0.041. Note: * indicates a significant path estimate at *p* < 0.05; ** indicates a significant path estimate at *p* < 0.01; *** indicates a significant path estimate at *p* < 0.001.

Overall, Table 6 shows that the theoretical model fits well (*χ^2^* = 448, *p* = 0.000, *df* = 245). Table 6 lists the test results of the model fit test.

The relationships between the latent constructs (research hypothesis) are tested based on the results of the theoretical model parameter estimation. The results are presented below.

(1) Continuous improvement has a significant positive impact on passenger perceived value (*β* = 0.83, *p* = 0.00), leading to Hypothesis 1 being accepted. This finding is consistent with customer perceived value theory. Continuous improvement of public car-sharing platforms is oriented to enhancing passenger enjoyment of the services. Public car-sharing platforms improve the economic value, utility value, emotional value, and social utility of the service through continuous improvement. These improvements are delivered to passengers in the service process. Passengers feel these improvements and perceive more value for the service. When the continuous improvement of public car-sharing platforms shortens a service process or it optimizes a certain service link, the perceived passenger value will increase significantly.
(2) The passenger perceived value has an important, positive impact on passenger loyalty (*β* = 0.46, *p* < 0.01), supporting Hypothesis 2. Passenger loyalty is developed from the perceived value of passengers and it is cultivated over time. Passengers face a variety of options, and the internal and external environment of public car-sharing platforms constantly changes. Passengers gradually develop loyalty when they constantly perceive the value of the service to be superior, and when they believe the service is better than other options.
(3) Passenger perceived value has a significant positive impact on passenger trust (β = 0.92, *p* = 0.00), and passenger trust has a clear positive impact on passenger loyalty (*β* = 0.20, *p* < 0.05). These results support Hypothesis 3 and Hypothesis 4. Passenger trust indicates that passengers believe the services that are provided by public car-sharing platforms can meet passenger needs. This means the service quality of public car-sharing platforms is stable, and public car-sharing platforms will not infringe passenger rights. Passenger trust is established over multiple transactions. After each transaction, the passengers will perceive and evaluate the value of the service. When the passenger perceived value is maintained at a high level or if it is continuously improved, the passenger will consider the service valuable. Passenger perceived value is a subjective feeling, and passenger trust is the subjective intention of the passenger. Both aspects fall within the scope of psychology.
  Passenger trust is the basis of passenger loyalty, and passenger loyalty is a manifestation of passengers’ deeper trust. Passengers trust the public car-sharing platform service and then choose the platform when using a shared car. Without this trust, passengers may be worried about the poor usage environment, a failure in the shared car used or the leakage of personal information. In this case, passengers would not use the service of the public car-sharing platforms.
(4) Passenger perceived value has a significant negative impact on transaction costs (*β* = −0.17, *p* < 0.05), and transaction costs has a negative impact on passenger loyalty (*β* = −0.05, *p* < 0.05). These results support Hypothesis 5 and Hypothesis 6. The perceived value of passengers is partially related to transaction costs, as passengers will compare the services received and costs paid. The higher the passenger’s perception of the service’s economic value, utility value, emotional value, and social utility, the more valuable the transaction costs paid by the passenger is, and the more inclined the passenger is to pay these costs.
  The "reduction" of passengers’ psychological transaction costs supports the formation of passenger loyalty. First, passengers do not need to search for alternative information regarding other public car-sharing platforms. Second, after the passengers have used the public car-sharing platform, it becomes easier to obtain platform information. As public car-sharing platform information will be published in a timely manner on different platforms, it will actively push the latest information to passengers. The coefficients of the two paths were significant; however, the path factor is very small. This might be because the transaction costs of the passengers using public car-sharing platforms is not high and is negligible when compared with transactions between business-to-business platforms.
(5) Unexpectedly, passenger trust has a very significant positive impact on transaction costs (*β* = 0.71, *p* < 0.01), thus refuting Hypothesis 7. In general, after passenger trust forms, transaction costs should be reduced. However, in this study’s survey of public car-sharing platforms, passenger trust has a positive impact on transaction costs. This might be because public car-sharing is new and it is still in the development stage. Passengers use shared cars less frequently, and passenger trust is only a short-term construct. When passengers use public car-sharing again, they need to recollect information on using it. It is also possible that in the initial stage, in order to increase the platforms usage rate, public car-sharing platforms issue large numbers of coupons and discount coupons. Later, after passenger trust has been formed, these preferential activities are no longer provided; consequently, passenger trust has a positive impact on transaction costs.

### 4.3. Direct, Indirect, and Total Effect Analysis

Table 7 summarizes the direct impact (*a_j_*), indirect impact (*b_j_*), and total impact (*c_j_*) of continuous improvement, passenger perceived value, passenger trust, and transaction costs on passenger loyalty.

The overall impact on passenger loyalty is as follows: continuous improvement (*c_1_* = 0.516), passenger perceived value (*c_2_* = 0.621), passenger trust (*c_3_* = 0.162), and transaction costs (*c_4_* = −0.054). Transaction costs have the least impact on passenger loyalty, perhaps because passengers using public car-sharing already require little learning effort. In the information age, it is easy to collect information and it does not require much effort or cost. This cost is very low when compared to business-to-business context (e.g., cooperation between companies). Passenger trust has a slightly higher impact on passenger loyalty than transaction costs. Passenger trust does not have a high impact on passenger loyalty, because passenger trust is short-term and segmental. In a public car-sharing platforms environment, this trust is easily transferred from a car-sharing platform to another car-sharing platform.

Continuous improvement and passenger perceived value have a high impact on passenger loyalty; passenger perceived value has the highest impact. Continuous improvement is completely indirect. That is, continuous improvement does not directly affect passenger loyalty, but it indirectly affects passenger loyalty through passenger perceived value, passenger trust, and transaction costs. The direct impact of passenger perceived value (*a_2_* = 0.462) is higher than its indirect impact (*b_2_* = 0.159). This shows that the passenger perceived value mainly directly affected passenger loyalty. Of all the direct impacts, the perceived value of passengers has the greatest impact on passenger loyalty (*a_2_* = 0.462).

### 4.4. Multi-Group Analysis

The estimation results of model parameters may differ across different sample groups. This study uses the multi-group structural equation modeling method to analyze the path differences between the two groups: those with a driver’s license and those with usage experience. A multi-group SEM analysis test is used to evaluate whether a model can be invariantly applied across sample groups.

In this study, the unstandardized regression weight table and the critical ratio matrix of each group in the AMOS results are imported into the Stats tool (downloaded from Gaskination’s StatWiki. [83]). This allowed for the generation of multi-group analysis results.

In the multi-group analysis model, the critical ratio between the parameters (z-Score) would be less than 1.96 if there were two corresponding parameters with the same attributes. This indicates that the two parameters can be considered to be equal. When the critical parameter ratio (z-Score) is greater than 1.96 and less than 2.58, the path coefficient values of the two groups differ at a 0.05 significance level. The critical parameter ratio (z-Score) is greater than 2.58, but less than 3.29 indicates that the path coefficient values of the two groups differ at the 0.01 significance level. If the critical parameter ratio (z-Score) are to be greater than 3.29, the path coefficient values of the two groups would be different at a 0.001 significance level.

#### 4.4.1. Multi-Group Analysis of Driver’s License

As mentioned in the introduction, the group with the driver’s licenses refers to the people who drive shared cars; in contrast, the group without driver’s licenses refers to the people who use (ride) shared cars.

The results of the multi-group analysis (see Table 8) show the effect of continuous improvement on passenger perceived value is greater in the user group with driver’s licenses (*β* = 0.865, *p* = 0.000) when compared to the user group without driver’s licenses (*β* = 0.787, *p* = 0.000). 

The change in path effect is at a significant level (*z*= −2.858 **). Therefore, a driver’s license (or the status as being a driver) has a moderating effect on the path from continuous improvement to perceived value. The degree of influence in the group with driver’s licenses is stronger than without driver’s licenses. The moderating effects of driver’s license (or driver status) on other paths are not significant.

#### 4.4.2. Multi-Group Analysis of Usage Experience

The group with experience (also called experienced users) is defined as passengers who have driven/used a shared car. The group without experience (also called inexperienced users) is defined as passengers who have not driven/used a shared car.

The results of the multi-group analysis (see Table 9) show that the impact of passenger perceived value on passenger trust is greater among experienced users (*β* = 0.987, *p* = 0.000) than for inexperienced users (*β* = 0.951, *p* = 0.000). The change in the path coefficient reaches a significant level (*z* = −1.978 *). Therefore, the experience of using the shared car has a moderating effect on the path of passenger perceived value on passenger trust. The degree of influence is stronger in the experienced user group when compared to the inexperienced user group.

The effect of transaction costs on passenger loyalty is greater for experienced users (*β* = −0.133, *p* < 0.01) as compared to inexperienced users (*β* = −0.045, *p* < 0.05); the path coefficient of the change reaches a significant level (*z* = −2.141 *). Therefore, the user experience has a moderating effect on the path from transaction costs to passenger loyalty, and the degree of influence is stronger for experienced users when compared to inexperienced users. There is no significant moderating effect of user experience on the adjustment of other paths.

## 5. Conclusions

This study integrates the theory of perceived value, trust theory, and transaction cost theory to propose a structural equation model that evaluates the impact of continuous improvement of public car sharing platforms on passenger loyalty. A multi-group structural equation model is used for moderation analysis. The research shows that the theoretical model is consistent with the real-world situation and it can be effectively used to reflect the relationships between potential constructs. The results of the structural equation modeling method show that passenger perceived value has the greatest direct impact on passenger loyalty. As such, public car-sharing platforms should prioritize passenger perceived value and provide valuable services to passengers. Continuous improvement only indirectly impacts passenger loyalty. Therefore, when developing a continuous improvement strategy, public car-sharing platforms should be guided by improving the passenger perceived value and should implement measures that reflect innovative service value.

Passenger trust has direct and indirect impacts on passenger loyalty. Therefore, public car-sharing platforms should provide passengers with reliable and stable service and solve the problems that passengers encounter in a timely way. This should strengthen the emotional connection with passengers and enhance passenger trust.

Although transaction costs directly impact passenger loyalty, the impact is minimal. However, public car-sharing platforms should also strive to reduce the transaction costs. Transaction costs form the most intuitive experience for passengers; reductions in transaction costs should significantly increase passenger use. Lower transaction costs also attract passengers who usually use taxis or other transportation methods.

The results of the multi-group analysis show that the influence of continuous improvement ability on passenger perceived value is more significant among users with a driver’s license than those without a driver’s license. The advantage of shared cars over buses, subways, and taxis is that they are more comfortable and cheaper. It is a good choice for young people that have a driving license without a private car. When compared with users that can only ride in shared cars without a driver’s license, they can also experience the joy of driving. The platforms can provide recreational methods, such as free books and game consoles in the car, in order to improve the experience of users without a driver’s license. The platforms should also allow users without a driver’s license to book a car as a rider, rather than only allowing drivers to book a car.

For inexperienced users, the effects of perceived value of passengers on passenger trust and transaction costs on passenger loyalty are significantly greater enhanced among experienced users. People who have never used a shared car cannot appreciate the value of shared cars, nor can they realize the cost reduction. This shows that the public car platform’s publicity and marketing efforts are not as effective in informing inexperienced users. Public car-sharing platforms should encourage people who have used car-sharing to recommend it to their relatives and friends. Perhaps the public shared car platform should offer free trails for inexperienced users.

Like all studies, this study has certain limitations. First, this study only examines the relationship between continuous improvement and passenger loyalty from three theoretical perspectives: perceived value theory, trust theory, and transaction cost theory. Future research can investigate this issue from other theoretical perspectives. It is recommended that future research incorporates the relative attractiveness, ease of use, and perceived usefulness into the impact of continuous improvement of shared car platforms on passenger loyalty. Second, since the data were collected from 31 provinces in mainland China, multi-group analysis by province is beyond the scope of this study. In the future, it is advisable to comprehensively obtain research data of eastern, central, and western China for multi-group analysis. This could provide corresponding recommendations that are related to decision-making on public car-sharing development in different regions. Third, perceived sacrifice is needed to test the perceived value. The survey did not measure perceived sacrifice. Future research should consider the effects of perceived sacrifice on perceived value and passenger loyalty. Regardless, this study provides a reference for the research on passenger loyalty of public shared cars, and a direction for the continuous improvement practice of public car-sharing platform companies.

## Figures and Tables

**Table 2:** Sample information.

Items	Type	Frequency	Percentage
Gender	Male	389	51.5%
	Female	366	48.5%
Age	18–25	458	60.7%
	26–35	151	20.0%
	36–45	100	13.2%
	46–54	44	5.8%
	≥ 55	2	0.3%
Driver’s license	Yes	337	44.6%
	No	418	55.4%
Education	≤ Senior	159	21.1%
	Specialist	98	13.0%
	Bachelor	477	63.2%
	Postgraduate	21	2.8%
Experience using car-sharing	Yes	181	24%
	No	574	76%

**Table 3:** Validity and reliability analysis.

Construct	Measure	*λ*	*α*	AVE	CR
Continuous Improvement	CIC1	0.790	0.784	0.613	0.905
	CIC2	0.772			
	CIC3	0.740			
	CIC4	0.787			
	CIC5	0.844			
	CIC6	0.760			
Passenger Perceived Value	PPV1	0.810	0.715	0.635	0.874
	PPV2	0.807			
	PPV3	0.815			
	PPV4	0.753			
Passenger Trust	PT1	0.728	0.776	0.601	0.883
	PT2	0.781			
	PT3	0.832			
	PT4	0.789			
	PT5	0.742			
Transaction Costs	TS1	0.808	0.761	0.606	0.860
	TS2	0.746			
	TS3	0.751			
	TS4	0.806			
Passenger Loyalty	PL1	0.831	0.766	0.621	0.867
	PL2	0.808			
	PL3	0.726			
	PL4	0.784			

**Table 4:** Average Variance Extracted and Squared Correlations of Constructs.

	CI	PPV	PT	TC	PL
CI	0.61 ^a^	0.18 ^c^	0.08	0.16	0.03
PPV	0.43 ^b^	0.64	0.27	0.02	0.04
PT	0.28	0.52	0.60	0.24	0.06
TC	0.40	−0.14	0.49	0.61	0.07
PL	0.16	0.20	0.25	−0.27	0.62

Notes:^a^ Average variance extracted values are along the main diagonal. ^b^ Correlations between constructs are below the main diagonal. ^c^ Squared correlations between constructs are above the main diagonal.

**Table 5:** Comparison between Alternative and Theoretical Models.

Model	*χ^2^*	*df*	Nested Model Comparison	△ *χ^2^*	sig.△ *χ^2^*	Decision
Alternative Model 1 (MA1)	444.16	242				
Alternative Model 2 (MA2)	445.27	243	MA1–MA2	1.11	*p* > 0.05	reject MA1 accept MA2
Theoretical Model (MT)	448.19	245	MA2–MT	2.92	*p* > 0.05	reject MA2 accept MT

**Table 6:** Fitting index and criterion.

	*χ^2^*/*df*	CFI	TLI	RMSEA	SRMR
Criteria	1–3	>0.90	>0.90	<0.08	<0.05
Value in this study	1.829	0.962	0.945	0.060	0.041

**Table 7:** Direct, indirect and total effects of antecedent variables on passengers’ loyalty.

Predictors.	Direct Effect	Indirect Effect	Total Effect
(j)	*(a_j_)*	*(b_j_)*	*(c_j_)*
Continuous improvement （j = 1）	-	0.516	0.516
Passenger Perceived Value （j = 2）	0.462	0.159	0.621
Passenger Trust （j = 3）	0.200	−0.038	0.162
Transaction Costs （j = 4）	−0.054	-	−0.054

**Table 8:** Path coefficients between two groups with and without driver’s licenses.

Path.	With Driver’s License		Without Driver’s License		*z*-Score
	Estimate	*p*	Estimate	*p*	
CI→PPV	0.865	0.000	0.787	0.000	−2.858 **
PPV→PL	0.584	0.047	0.591	0.042	−0.182
PPV→PT	0.929	0.000	0.915	0.000	1.335
PT→PL	0.313	0.036	0.274	0.045	0.067
PPV→TC	−0.197	0.042	−0.169	0.034	0.174
TC→PL	−0.082	0.047	−0.055	0.031	0.555
PT→TC	0.721	0.057	0.683	0.064	−0.200

Notes: ** *z*-Score < 0.01.

**Table 9:** Path coefficients between groups with and without user experience.

Path	With Experience		Without Experience		*z*-Score
	Estimate	*p*	Estimate	*p*	
CI→PPV	0.938	0.000	0.873	0.000	−0.701
PPV→PL	0.526	0.003	0.510	0.006	0.322
PPV→PT	0.987	0.000	0.951	0.000	−1.978 *
PT→PL	0.175	0.036	0.145	0.045	0.276
PPV→TC	−0.160	0.049	−0.187	0.024	0.022
TC→PL	−0.133	0.047	−0.045	0.031	−2.141 *
PT→TC	0.632	0.058	0.652	0.061	−0.16

Notes: * *z*-Score < 0.05.
